# Not all poor are equal: the perpetuation of poverty through blaming those who have been poor all their lives

**DOI:** 10.1007/s12144-022-03804-6

**Published:** 2022-10-05

**Authors:** Joaquín Alcañiz-Colomer, Miguel Moya, Inmaculada Valor-Segura

**Affiliations:** 1 Department of Social Psychology, University of Granada, Campus de Cartuja, s/n, 18071 Granada, Spain; 2 Research Centre in Mind, Brain, and Behavior (CIMCYC), Granada, Spain

**Keywords:** Poverty perception, Public policies attitudes, Attributions for poverty, Deservingness, Economic crisis, Identification

## Abstract

The economic crisis of 2008 severely affected the welfare states. As the economic situation of a country worsens, the resources that the public administration can devote to improve the situation of the people also decrease, endangering the advancement of those in a disadvantaged situation. People who have always lived in poverty, besides having their opportunities reduced, also face negative public views that affect the perceived legitimacy of such public aid, which can in turn be a mechanism for perpetuating their situation. Two studies (*N* = 252 and *N* = 266) analyse how a person in persistent poverty is perceived compared to a person in poverty due to the crisis—a circumstantial poverty. We also study some feasible mechanisms underlying this different perception, as well as their effects on attitudes toward social protection policies. In Study 1, results indicated that people showed more favourable attitudes toward social protection policies when they perceived someone in poverty due to the crisis, compared to the target who had been in poverty all his/her life. Individualistic attributions for poverty mediated this effect: when people think of someone in persistent poverty, they make more individualistic attributions concerning their situation, which leads to worse attitudes toward social protection policies. Identification with the group moderates this relation. Furthermore, Study 2 showed that participants perceive people who are in poverty because of economic crisis as more deserving of help than people who have always been poor. Some theoretical and practical implications for intergroup relations and public policy are discussed.

**Supplementary Information:**

The online version contains supplementary material available at 10.1007/s12144-022-03804-6.

Poverty and inequality not only persist due to inefficient economic systems or lack of resources for people in poverty, but also because they are a product of human relationships, which determine how resources are distributed (Lemieux & Pratto, 2003). People in persistent poverty, who have lived this as a permanent and reproducible phenomenon, are at the bottom of the social hierarchy and have less access to resources and opportunities (Rucker et al., 2018). Here, we explore some mechanisms that may perpetuate this situation; specifically, we focus on attitudes towards social protection policies and how they are affected by attributions for poverty, deservingness of social protection, and stereotypes, comparing the perception of the aforementioned group with people made poor in the wake of economic crisis—which can be considered as a situation of circumstantial poverty. We also consider the role of the participants’ group membership in the perception of different types of poverty.

## Different types of poverty in times of economic crisis and social protection policies

In Spain, during the last economic crisis the number of unemployed people soared from 1,806,200 million (an unemployment rate of 8.1%) in the third quarter of 2007 to the highest peak of 5,943,400 million (an unemployment rate of 25.65%) in the third quarter of 2013 (Instituto Nacional de Estadística, 2007-2013). The average unemployment rate in the previous years was 10.05 (Instituto Nacional Estadística, 2001-2006). Based on European Community Household panel data, the average number of Spanish households below the poverty line was 18.8 between 1994 and 2000 (Instituto Nacional Estadística, 1994-2001). This indicator, the poverty risk rate, increased from 19.8% in 2007 to 22.2% in 2014 (Instituto Nacional de Estadística, 2007-2014). The deterioration of the labor market implies an increase in the heterogeneity of the group of people with few resources. It contains both people who have lived through poverty as a permanent and reproducible phenomenon, in addition to declassed people who have lost their social status or have been victims of difficulties that they did not encounter before (Paugam, 2007). The crisis also led to austerity policies in most countries, which reduced investments in social policies, something especially important in Spain (Guillén et al., 2016).

The consequences of the crisis have been devastating for many people, but this reality could be eclipsing a tougher one: the life doomed to poverty of many who have not known another situation. We face a theoretical vacuum that may have important practical implications, because public opinion influences policies to guarantee the welfare of the most disadvantaged people (Brooks & Manza, 2006; Burstein, 1998; Myles, 2006; Stimson et al., 1995). Furthermore, people who have been in poverty all their lives might need different social protection policies by comparison with those who are poor due to economic crisis. For example, the former need social protection measures focused on their income needs and not in their work history, insofar as these households have different characteristics (e.g., Cantó et al., 2012).

Social protection policies are one of the foundations of the welfare state, hence the importance of public opinion on welfare policies in general and poverty assistance in particular. The incorporation of these policies into the sphere of political priorities will depend on the perception by politicians that, in doing so, they will gain the approval of their electorate for their management (Luhmann, 1990). Public opinion legitimizes which populations are seen as deserving of the social policies that protect them, which in turn influences support for the welfare state itself (van Oorschot, 2010). Previous research has shown that attitudes towards social protection are influenced by several variables including self-interest (Hasenfeld & Rafferty, 1989), ideological preferences (Armingeon & Weisstanner, 2021), the perception of whether people deserve their poverty status (Appelbaum, 2001), and causal attributions about the origin of poverty (Alston & Dean, 1972; Bullock et al., 2003; Piff et al., 2020).

Crucially, the group target of social protection policies also influences the support shown towards it (Appelbaum, 2001; van Oorschot, 2000). Appelbaum (2001) showed that people were more favourable to recommend liberal policies (e.g., full medical coverage with no time limits) to some groups of the needy (e.g., physically handicapped people) in comparison with others (e.g., able-bodied men), being the former group seen as more deserving and less responsible.

## Causal attributions of poverty and help deservingness

Beliefs about the nature of poverty affect several attitudes related to the difficulties in eradicating it. People can attribute poverty to various causes, mainly: a) individualistic, that is, to the person in poverty; b) structural causes, such as external, social and structural forces; c) fatalistic causes, where the responsibility for poverty lies with factors such as illness or bad luck (Feagin, 1972; Feather, 1974). Other more detailed categorizations are possible, but in this article we are interested in comparing structural attributions with individualistic ones. Individualistic attributions involve factors related to people in poverty themselves, such as their lack of will or lack of capacity, which implies making them responsible for their situation. Structural attributions stress factors beyond the person’s own control, such as lack of jobs to access or low wages, thus not placing the responsibility for the person’s situation on them.

These attributions may vary depending on the perceived group of people in poverty. For example, Henry et al. (2004) found that individualistic attributions are more likely for welfare recipients relative than for poor people. Previous research has found that preferences for structural explanations of poverty predicts support for progressive welfare programs, while individualistic attributions predict support for restrictive welfare programs (Bullock et al., 2003). As we stated above, the group that will be the object of such assistance influences the preference for progressive compared to restrictive welfare programs; for example, it is more likely that liberal policies will be recommended (e.g., cash benefits and no-cash benefits with no time limits) for widows with children, who are seen as less responsible of their situation, than for able-bodied men, who can be seen as more responsible (Appelbaum, 2001). In addition to the correlational evidence, some studies have explored the causal influence of poverty attributions on other relevant variables related to poverty alleviation. For instance, Farwell and Weiner (2000) presented different vignettes where the responsibility of the person in need for their situation was manipulated (responsible vs. nonresponsible) and participants were asked how much financial assistance they would recommend for each character. Their results showed that participants recommended more funds when the person in need was not responsible for their situation. A similar reasoning, explaining financial support to people in need in terms of responsibility for their situation, has been found in other studies (Skitka & Tetlock, 1992). According to the mentioned findings, we expect that people in persistent poverty will receive more individualistic attributions about their poverty, in comparison to people who are poor as consequence of the economic crisis.

While attributions for poverty are important in explaining support for social protection, there are other important factors related to the characteristics of benefit recipients that influence greater or lesser support for these policies, namely the extent to which they are considered deserving of social protection. Van Oorschot (2000), based on the literature in support for social protection policies in welfare states, raises five dimensions or criteria of “deservingness”: i) control, or responsibility of individuals for their own situation—the less control or responsibility, the greater the deservingness—; ii) need—the greater need perceived, the greater the deservingness—; iii) identity—the closer to “us”, the greater the deservingness—; iv) compliance—the more obedient, the more deserving—(e.g., if they follow the rules in a docile manner when applying for subsidies); and v) reciprocity, or the belief that they will do something in return for the help. For instance, Cook (1979) observed that people showed higher levels of support when recipients were considered to have a higher level of need and when they were perceived as not responsible for their situation. This perceived deservingness may vary depending on the characteristics of the person that would receive the help (Kootstra, 2016). Thus, we expect that people in persistent poverty will be perceived as less deserving of social protection policies, in comparison to people who are in poverty as consequence of the economic crisis.

## Group membership and attitudes towards people in poverty

Attitudes towards people in poverty and towards resources distribution also depend on some characteristics of the people who maintain such attitudes. One of these important characteristics is their membership’ group. Thus, for instance, middle-class people tend to make more individualistic attributions, blaming people in poverty for their situation, while social assistance beneficiaries tend to make more structural attributions (Bullock, 1999).

Another factor related to group membership is the stability with which such belonging is perceived. For instance, middle-class perceivers might identify more with people who have just fallen into poverty as consequence of the crisis -thinking that they could find in the same situation- and this identification led them to make less individualistic attributions about this group and perceive them as more deserving of social protection. Also is possible that the middle-class people seek to obtain a more positive identity by differentiating itself from those in permanent poverty.

## Stereotypes about people in poverty

Lastly, stereotypes play an important role in the perpetuation of inequality and poverty insofar as they help maintain the system by justifying a series of social actions (Tajfel, 1981). Stereotype Content Model (Glick & Fiske, 1999) postulate that there are two basic dimensions in stereotypes towards any group: competence and warmth. The first would be the ability to achieve goals and the second would be interpersonal sympathy. These two dimensions, supposedly, would have a universal character (Cuddy et al., 2007; Fiske et al., 2007), although some aspects of certain stereotypes show some cultural variations (Cuddy et al., 2009). In the case of people with low socioeconomic status, empirical evidence showed that they are seen as not very competent and with low warmth (Fiske et al., 2007), although other times they are seen as not competent but with higher levels of sociability compared to rich people (Durante et al., 2017; Durante et al., 2013). Cuddy et al. (2007) found that warmth stereotypes eliciting active facilitation (helping, protecting) whereas competence stereotypes were not related with these behavioral tendencies.

Empirical evidence from the Stereotype Content Model also shows differences among subgroups within people in poverty, such as homeless people, recipients of charity, or poor people in general (Fiske et al., 2007). For example, Fiske et al. (2007) found that people had a worse perception of homeless people than that they had of welfare recipients, that is, the former were seen as less competent and warm in comparison with the latter. In this research we also explored whether stereotypes about people in poverty due to economic crisis and people in persistent poverty are different.

## Overview of the present research

In these studies, we focused on examining the perceptions about people in persistent poverty compared to those of people in poverty as a result of an economic crisis as well as the influence that these perceptions may have on attitudes towards social protection. Previous studies have highlighted the importance of beliefs about the origin of poverty, or attributions for poverty, in attitudes towards social protection. With this emphasis in mind, we consider this variable fundamental in people’s perceptions of the aforementioned groups. Specifically, we propose that a differentiated pattern of attributions of these two groups’ situations will lead to different general attitudes towards social protection policies. We also will analyse the influence of deservingness perceptions, the participants’ identification with both groups, and the various stereotypes people apply to people in poverty shown in their attitudes towards social protection of both groups of people in poverty.

These studies make two contributions to the literature. First, they contribute to the literature by demonstrating that individuals’ attitudes towards social protection policies differ depending on which type of poor person is activated in their mind: the poor person due to the crisis o the poor person who was born and raised in poverty. Therefore, when social debates concern “the poor”, it is convenient to be clear about what kind of people in poverty we are talking about. If politicians, for instance, want citizens to support their policies of increasing aid to people in poverty, one tactic might be to make certain types of people in poverty (those whom people may perceive as more deserving of help) salient in the citizens’ minds. Second, analysing the importance of attributions for poverty in the perception of various groups of people in poverty, deservingness, and stereotypes as well as the participants’ identification with each group will help explain how people perceive poverty as well as the opposition to policies to eradicate poverty. This analysis will also show where we should intervene if we want to change these attitudes.

All datasets, measures, and preregistered forms of both studies are publicly available at https://osf.io/ne6ug/?view_only=af98fb36c3ef4eb98fa19542cde3467b.

## Study 1

In Study 1, we aimed to analyse the perception of the aforementioned two groups of people in poverty in terms the perceived causes of their poverty, stereotypes about them, identification with these groups, and attitudes towards social protection policies. We also include some measures of the objective and subjective social class, system justification ideology, social-dominance orientation, and financial threat with an exploratory purpose. We present the results concerning these additional variables in the supplementary materials.

### Method

#### Participants

We recruited a sample of 290 Spanish undergraduate students in university libraries at a university in Southern Spain. We excluded 38 participants because Spanish was not their native language or they failed the attention check. As noted in the preregistration, we attempted to recruit 260 participants to observe an effect size of *d* = 0.35 with a power of .80 and an alpha of .05. We determined this small to medium effect size taking into account our available resources and the necessary time to collect the sample. So this effect size was determined based on our availability of resources to obtain the sample and we relied on a benchmark. This may not be the best possible strategy, however, because we are not answering any theoretical questions (see Lakens et al., 2018). The final sample included 252 participants (152 women); their mean age was 21.98 (*SD* = 3.16; see Table 1 for descriptive information about the samples in both studies). In the final sample, 122 participants were assigned to the condition referring to a person in persistent poverty and 130 to the condition referring to a person in poverty due to an economic crisis. We performed a sensitivity analysis using G*Power 3.1 (Faul et al., 2007) to find differences between two independent groups (alpha level = .05, 80% power). This analysis, performed on the sample after we applied our exclusion criteria, suggested that we were able to detect an effect size *d* = 0.35.

**Table 1:** Descriptive Information about Samples in Study 1 and Study 2

Variable	Study 1		Study 2	
	n	%	n	%
Gender				
Men	100	39.7	117	44
Women	152	60.3	149	56
Not reported	–	–		
Income				
< 650	12	4.8	24	9
651-1.300	50	19.8	59	22.2
1.301-1.950	65	25.8	79	29.7
1.951-2.600	62	24.6	57	21.4
2.601-3.251	29	11.5	20	7.5
3.251-3.900	18	7.1	11	4.1
3.901-4.550	4	1.6	8	3
4.551-5.200	6	2.4	1	0.4
>5.200	5	2	5	1.7
Not reported	1	0.4	3	1
Participant education				
Primary School	3	1.2	1	0.4
Secondary education	–	–	7	2.6
Vocational Training	8	3.2	45	16.9
High School/Diploma	1	0.4	15	5.6
University not completed	187	74.2	95	35.7
University completed	29	11.5	65	24.4
Master’s degree	14	5.6	29	10.9
Doctorate	10	4	6	2.3
Not reported	–	–	3	1.1

#### Procedure and materials

Participants agreed to participate voluntarily in a study on poverty perception. We randomly assigned them to one of two possible conditions. In each condition, they had to read a text about a poor person. In one condition, this person became poor in the wake of an economic crisis, and in the other, the person had been poor all his life. Next, participants had to complete the measures listed below. All measures except for the group identification measure were generic, not specifically worded for each group of people in poverty in each condition.

##### Experimental manipulation

We asked the participants to read a short vignette about a character named Antonio. The text was similar in both experimental conditions, but the information about the duration and causes of his poverty was different. The text was as follows:

Antonio is from a city in eastern Spain. He’s married and has two children. Antonio has difficulty reaching the end of the month since he lost his job because of the crisis (has had difficulties reaching the end of month all his life). As for many other people, his loss of employment because of the crisis made him go from being in a financially comfortable situation to being considered poor (he was born and raised in a poor home). Since then (since he was old enough), he has been doing some occasional jobs to get some money or borrowing from his acquaintances to meet the needs of his household.

As an attention check, we required the participants to answer a question about the nature of the poverty concerning the person in the story, which they had read immediately beforehand (He has always been poor/He is poor in the wake of an economic crisis). This was our main independent variable in both studies.

##### Stereotypes about the poor

We measured stereotypes for the two targets along two core dimensions, competence (α = .83) and warmth (α = .78), based on the Stereotype -Content Model (Cuddy et al., 2007; Glick & Fiske, 1999). The participants evaluated the extent to which the target had some competence (five items; e.g., “competent,” “competitive”) and warmth (six items; e.g., “tolerant,” “trustworthy”) on a 5-point Likert scale (1 = *totally disagree* to 5 = *totally agree*) to indicate their degree of agreement with every item. The scale was preceded by an introduction that clarified that we sought to know the opinion of society, not of the concrete respondent, to avoid desirability bias.

##### Attributions for poverty

We measured this construct using an 18-item scale. The respondents answered on a 5-point Likert scale (1 = *totally disagree* to 5 = *totally agree*). We translated the items into Spanish from Furnham (1982) and Weiner et al. (2011), and added some items concerning the Spanish context (e.g., “People do not want to move to work in other locations”). The individualistic attributions subscale contained 10 items (α = .81; e.g., “Lack of effort or laziness”), and the structural attributions subscale contained eight items (α = .67; e.g., “Lack of opportunity”).

##### Group identification

We measured participants’ identification with our two groups of people in poverty by adapting to this context, changing the targets, the collective identity scale from Leach et al. (2008). The possible responses ranged from 1 (*totally disagree*) to 5 (*totally agree*). Specifically, we used items from the solidarity subscale and the individual self-stereotyping items that best fit our purpose. Our scale contained seven items (e.g., “I feel committed with poor people”) and was preceded by the next text: “Think of someone who has been poor all his/her life (is poor due to the economic crisis) and answer the following questions.” Subjects responded to these items twice, once referring to the group perceived in their experimental condition and once referring to the other group. We subtracted from the score on the identification scale regarding poor people in the wake of the crisis (α = .67) the score on the identification scale regarding people in chronic poverty (α = .71) to obtain a global identification index. Higher positive scores indicate a stronger identification with the poor in the wake of the crisis (versus the poor in chronic poverty).

##### Attitudes towards social protection policies (α = .76)

We measured this variable, our main dependent variable, on a 5-point Likert scale (1 = *totally disagree* to 5 = *totally agree*), with which subjects indicated their degree of agreement with 20 statements about social protection policies (e.g., “Many people on social benefits try to find work in order to be able to live on their own”). We used a scale inspired in Furnham (1985) scale—in Spanish in this study— referred to social policies in this study; in addition, we included some items about the Spanish context (e.g., “In general, little money is spent on social policies in this country”). Higher scores indicate a better general attitude towards social protection.

##### Political ideology

We measured political ideology using an item whose possible responses ranged from 1 to 10: “In politics, sometimes people talk about ‘left’ and ‘right.’ Using a scale where 1 means ‘extreme left’ and 10 ‘extreme right,’ where would you position yourself on this scale?” As we indicated in the Method section, we also included other scales. For space reasons, we provide the details in supplementary materials.

#### Preregistered hypotheses

We expected that compared to people in chronic poverty, those who were in poverty due to the economic crisis would receive less individualistic and more structural attributions (Hypothesis 1a) and would be perceived as more competent and warmer (Hypothesis 1b); we also predicted that attitudes towards social protection policies would be better when participants perceive someone in poverty due to the economic crisis relative to those in chronic poverty (Hypothesis 1c). For this group of hypotheses, our main independent variable was the experimental condition and attributions for poverty, competence, and warmth perceptions, and attitudes towards social protection policies were our dependent variables.

Identification with the group would moderate the relationship between the perceived group—person in chronic poverty versus person in poverty due to economic crisis—and the assignment of competence and warmth scores (Hypothesis 2) and attitudes towards social protection policies (Hypothesis 3). We expected that the tendency to assign greater competence and sociability to people in poverty in the wake of a crisis (versus people in persistent poverty) and to show a better attitude towards social protection policies when perceiving the former would be more intense among people who identify with those who are in poverty because of the economic crisis. Therefore, in this set of hypotheses, our independent variable was the experimental condition, the moderator variable was identification with the group, and the dependent variables were warmth and competence perceptions and attitudes towards social protection policies.

Finally, we expected that attributions for poverty would mediate the relationship between the perceived group (poverty due to economic crisis vs. persistent poverty) and support for social protection policies (Hypothesis 4), in the sense that perceiving people falling into poverty due to economic crisis would lead to fewer individualistic attributions for their poverty and more support for social protection policies.

### Results

#### Differences among causal attributions for poverty, attitudes towards social protection policies, and stereotypes about poor people

To test our first hypotheses, H1a, H1b, and H1c, we conducted a *t* test for differences between two independent means (see Table 3 for means and standard deviations of main variables). We found partial support for Hypothesis 1a because the hypothesised results were confirmed only for individualistic attributions, not for structural ones: Participants who had read about someone who has been poor all his life made more individualistic attributions than those who had read about someone who is poor due to the economic crisis, *t*(250) = −2.16, *p* = .03, BCa 95% CI [0.02, 0.38], *d* = 0.27. This effect size was smaller than the effect size that sensitivity analyses suggested we could detect, so we conducted a post hoc analysis using G*Power 3.1 (Faul et al., 2007) for differences between the two independent groups. We computed the observed effect size and sample size for each condition, and post hoc analysis revealed that the power achieved for this analysis was 0.63. Differences in structural attributions for poverty were not statistically significant, *t*(250) = −0.83, *p* = .41, BCa 95% CI [−0.18, 0.07], *d = −*0.10. Our findings partially supported Hypothesis 1b because the hypothesised results were confirmed only for the competence dimension. As we expected, the participants perceived the person in persistent poverty as less competent than the person in poverty due to an economic crisis, *t*(250) = 4.17, *p* < .001, BCa 95% CI [−0.56, −0.20], *d = −*0.53. We found no statistically significant differences in the warmth subscale scores, *t*(250) = −1.79, *p* = .08, BCa 95% CI [−0.33, 0.02], *d = −*0.22. Our findings supported Hypothesis 1c: Attitudes towards social protection policies were less favorable after reading a vignette about a person who has been in poverty all his life than after reading a vignette about person in poverty as a result of the economic crisis, *t*(250) = 3.19, *p* = .002, BCa 95% CI [−0.37, −0.09], *d = −*0.40. Table 2 presents correlations between variables in this study.

**Table 2:** Correlations Between Variables in Study 1

	1	2	3	4	5	6	7
1. Competence	–						
2. Warmth	.65**	–					
3. Individualistic attributions	−.03	−.03	–				
4. Structural attributions	.11	.03	−.07	–			
5. Social protection policies attitudes	.00	−.07	−.57**	.05	–		
6. Index identification	−.08	.01	.07	−.16	−.12	–	
7. Political ideology	.01	−.01	.41**	−.04	−.59**	.03	–

** *p* < 0.01 (2-tailed) * *p* < 0.05 (2-tailed)

#### Group identification as moderator in the relationship among perceived group, stereotypes, and attitudes towards social protection policies

We tested Hypotheses 2 and 3 using the PROCESS macro for SPSS, Model 1, with 10,000 bootstrapped samples. To test Hypothesis 2, we included the experimental condition as the independent variable and the global identification index as the moderator variable. We built two models. In one model, the criteria variable was competence scores and in the other one, warmth scores. We used the same analytical strategy to test our hypotheses in this case, including attitudes towards social protection policies as a dependent variable.

We did not find support for Hypothesis 2, that group identification will moderate the relation between perceived group and assignment of competence and warmth. For warmth, the interaction effect of poverty condition ✕ group identity was *b* = 0.20, *t*(248) = 1.96, *p* = .051, 95% CI = [−0.00, 0.41], and for competence, this interaction effect was *b* = 0.20, *t*(248) = 1.87, *p* = .062, 95% CI = [−0.01, 0.42]. As we hypothesized in Hypothesis 3, group identification moderated the relationship between perceived group and attitudes towards social protection policies, *b* = 0.25, *t*(248) = 2.95, *p* = .003, 95% CI = [0.84, 0.42]. Perceiving someone in poverty due to economic crisis (compared to someone in persistent poverty) increased positive attitudes towards social protection policies among those who identified more with the first group (understood as a standard deviation above the mean score on the identification index), *b* = 0.44, *SE* = 0.10, *p* < .001, 95% CI = [0.24, 0.65]; among those who identified less with people the economic crisis made poor, the effect was nonsignificant (understood as a standard deviation below the mean score on the identification index), *b* = 0.01, *SE* = 0.10, *p* = .91, 95% CI = [−0.16, 0.22].

#### The mediating role of attributions for poverty in the relationship among perceived group, attitudes towards social protection policiessocial-protection policies, and stereotypes

We tested Hypothesis 4 by conducting a linear regression analysis using the PROCESS macro for SPSS, testing mediation Model 4. All confidence intervals for indirect effects are a BCa CI based on 10,000 bootstrapped samples. We found partial support for Hypothesis 4 because the hypothesised results were confirmed only for individualistic attributions, not for structural ones. Therefore, we found a significant indirect effect of the perceived group on the attitudes towards social protection policies, through individualistic attributions for poverty, *b* = 0.09, BCa 95% CI [0.01, 0.17]. In this model, the direct effect was *b* = 0.14, *p* = .01 and the total effect *b* = 0.23, *p* = .002; the perceived condition’s effect on individualistic attributions was *b* = −0.20, *p* = .03, and the effect of the latter on attitudes towards social protection policies was *b* = −0.44, *p* < .001. Following Wen and Fan’s (2015) recommendations regarding monotonic indices, we reported the proportion of indirect effect relative to the total effect (*P*_*M*_) together with the total effect. We calculated this measure using the lavaan (Rosseel, 2012) R package (R Core Team, 2017), with 10,000 bootstrap replications, BCa method. In this case, *P*_*M*_ was 0.38, BCa 95% CI [0.04, 0.76]. The indirect effect remained significant, even when we controlled for political ideology. Structural attributions did not play a significant mediator role in the relationship between the type of poor person perceived and attitudes towards social protection policies, *b* = 0.00, BCa 95% CI [−0.01, 0.02]. We controlled for participants’ political ideology, considering that previous research shows that conservatism is an important factor affecting poverty attributions (e.g., Zucker & Weiner, 1993), and it remained statistically significant. In addition, we built a model to test Hypotheses 3 and 4 together (see Fig. S1 in Supplementary Information). This model yielded results quite similar to those of testing the hypotheses separately; however, the interaction between the experimental condition and group identification was no longer significant (*b* = 0.16, *p* = .054) although the analysis of the simple lines showed the same pattern we previously found. Individualistic attributions remained a significant mediator between experimental condition and attitudes towards social protection policies.

### Discussion

Our results showed that attitudes towards social protection policies differ depending on the nature of the perceived target’s poverty: people who have been in poverty all their lives versus people in poverty in the wake of economic crisis. Individualistic poverty attributions mediated the relationship between perceived group and attitudes towards social protection policies. When participants perceived someone who has been poor all his life, they made more individualistic attributions, which led to a worse attitude towards social protection policies (in comparison to those who perceived someone who is poor due to an economic crisis). Considering the studies that causally link individualistic attributions with lower support for the provision of funds to people in poverty (e.g., Farwell & Weiner, 2000), our proposed causal model seems the most plausible. Our results are also consistent with those of recent studies, which have shown a causal relationship between individualistic poverty attributions and attitudes towards redistribution (Bai et al., 2022).

The person in persistent poverty was seen as less competent although the perception of the targets in terms of competence and warmth did not significantly affect the attitudes towards social protection policies targeted to both groups. Identification with the group was a significant moderating variable: Participants who identify more with people who became poor in the wake of an economic crisis show a better attitude towards social protection policies when presented with this group. However, when we tested all the effects in a single model, this effect was no longer significant, so this variable’s ability to explain attitudes towards social protection must be nuanced.

A possible limitation of the present study is the sample—college students in this case. Therefore, in Study 2, we sought to examine these findings’ robustness in a general-population sample. As we previously theorized, we included other variables, such as the perceived deservingness of social protection, that may help shed light on this relationship between the perceived person and attitudes towards social protection policies.

## Study 2

To replicate the results from Study 1 in a general-population sample and to explore perceived deservingness’s effect on social protection, we used the same experimental manipulation and most of the measures presented in Study 1. We included measures apart from those reported here (objective and subjective social class and social-dominance orientation); we present results concerning these measures in the supplementary materials. As we did in Study 1, and following the same rationale, we controlled for political ideology. In this study, we attempted to recruit 300 participants to increase the statistical power to test our hypothesis about mean differences, taking into account the number of participants excluded from the first study. However, we were not able to collect more than 280 observations. We also performed a sensitivity analysis using G*Power 3.1 (Faul et al., 2007) in this study for differences between the two independent groups (alpha = .05, 80% power). Sensitivity analysis of the sample after exclusions suggested that we were able to detect an effect size *d* = 0.34.

### Method

#### Participants

We recruited a sample of 280 participants from the Spanish general population at a public-transport station. Participants agreed to participate voluntarily, without any compensation, in a study on poverty perception, and then we randomly assigned them to one of two possible conditions. As in the previous study, in each condition, they had to read a text about a poor person. In one condition, this person became poor in the wake of an economic crisis, and in the other condition, this person had been poor all his life. Next, participants had to complete other measures, listed below. After we applied our exclusion criteria, 266 participants (149 women) remained; their mean age was 30.55 (*SD* = 11.85; see Table 1 for descriptive information about the sample). In the final sample, 130 participants completed the questionnaire about a person in persistent poverty and 136 answered the questionnaire about a person in poverty due to an economic crisis.

#### Procedure and materials

As in Study 1, participants agreed to participate voluntarily in a study on poverty perception without obtaining any economic or other type of reward. After they agreed to participate, we assigned them to one of two possible experimental conditions and asked them to respond to the measures presented here.

##### Experimental manipulation

The text they had to read was the same as that described in Study 1 except for the information about the target’s marriage and children, which we eliminated to control for distortion’s possible effect on the perceived deservingness of social protection, as we know that this information can affect it (e.g., Will, 1993).

##### Dependent variables and covariates

We used the same measures as in Study 1: individualistic poverty attributions (α = .85) and structural attributions (α = .75), a group identification index (subtracting from the score on the identification scale with poor people in the wake of the crisis (α = .78) the score on the identification scale with people in chronic poverty (α = .80), attitudes towards social protection policies (α = .89), and political ideology (see Table 3 for means and standard deviations of main variables in this study).

**Table 3:** Means and Standard Deviations for Main Measures in Study 1 and Study 2

	Study 1		Study 2	
	Condition		Condition	
	Persistent poverty	Poverty due to economic crisis	Persistent poverty	Poverty due to economic crisis
Competence	2.17^a^ (0.69)	2.55^b^ (0.76)		
Warmth	2.95^a^ (0.72)	3.11^a^ (0.66)		
Individualistic attributions	2.58^a^ (0.83)	2.38^b^ (0.61)	2.53^a^ (0.78)	2.05^b^ (0.65)
Structural attributions	3.02^a^ (0.58)	3.07^a^ (0.44)	3.01^a^ (0.65)	3.21^b^ (0.47)
Identification index	0.35^a^ (0.90)	0.45^a^ (0.78)	0.29^a^ (1.16)	0.72^b^ (0.99)
Attitudes toward social protection policies	3.24^a^ (0.59)	3.47^b^ (0.56)	3.16^a^ (0.64)	3.54^b^ (0.53)
Deservingness of social protection			3.64^a^ (0.70)	4.06^b^ (0.54)
Political Ideology	3.80^a^ (1.97)	3.72^a^ (1.78)	4.10^a^ (1.97)	4.04^a^ (1.95)

Standard deviations are presented within parentheses. Within the same study, rows with a different superscript differ at *p* < .05

##### Perception of social protection deservingness (α = .87)

We measured this variable using a 10-item scale, in which the participants responded on a 5-point Likert scale (1 = *totally disagree* to 5 = *totally agree*), with which subjects indicated their degree of agreement with statements about poor people’s deservingness of social protection. The scale is based on van Oorschot’s (2000) work. Higher scores indicate greater perceived deservingness of social protection policies. Van Oorschot (2000) initially postulated five dimensions (control, need, identity, attitude, and reciprocity). We decided to include only the dimensions of need (three items; e.g., “people in that situation usually have a great need to be helped”), attitude (four items; e.g., “people in that situation appreciate being supported”), and reciprocity (three items; e.g., “the state can help people in that situation, and these people will somehow return to the state what they have received”). We excluded group identity and control over their situation because we believe they are redundant in our scales of identification and individualistic attributions for poverty. A factor analysis showed the existence of only one factor with eigenvalues greater than 1 (4.10), which explained 41% of the variance. Therefore, we calculated the total score of all the items included in the three dimensions. We also included other scales, but they are not relevant for the purpose of this study. For space reasons, we present the details in the Supplemental Materials.

#### Preregistered hypotheses

We expected that compared to people in chronic poverty, those who are in poverty due to an economic crisis would receive less individualistic and more structural attributions (Hypothesis 1a) and would be perceived as more deserving (Hypothesis 1b). Although the differences in structural attributions were not significant in Study 1, we preregistered this hypothesis again because we thought that perhaps the absence of effects occurred due to the nature of the sample (college students in Study 1). Participants who perceive someone in poverty due to an economic crisis would also have better attitudes towards social protection policies (Hypothesis 1c). Our independent variable for these hypotheses was the experimental condition. Attributions for poverty, deservingness of social protection, and attitudes towards social protection were the dependent variables.

Identification with the group would moderate the relationship between the perceived group—person in chronic poverty versus person in poverty due to economic crisis—as well as perceived deservingness of social protection (Hypothesis 2) and attitudes towards social protection policies (Hypothesis 3). We expected that the tendency to assign greater deservingness to people in poverty in the wake of the crisis (versus people in persistent poverty) and to show a better attitude towards social protection policies when perceiving the former would be more intense in people who identify with those who are in poverty because of an economic crisis. Again, in these hypotheses, our independent variable was the experimental condition, the moderating variable was identification with the group, and the dependent variables were perceived deservingness of social protection and attitudes towards social protection policies.

Finally, attributions for poverty would mediate the relationship among the perceived group (poverty due to economic crisis versus chronic poverty), perceived deservingness of social protection (Hypothesis 4), and attitudes towards social protection policies (Hypothesis 5).

### Results

#### Differences among causal attributions for poverty, attitudes towards social protection policies and deservingness of social protection

We conducted a *t* test to identify differences between the two independent means (two groups) to test our first hypotheses: H1a, H1b, and H1c. Table 3 presents the mean scores. Our findings fully supported Hypothesis 1a. Replicating Study 1’s findings, participants who perceived someone who has been poor all his life made more individualistic attributions than those who perceived someone made poor due to an economic crisis, *t*(264) = 5.43, *p* < .001, BCa 95% CI [0.31, 0.65], *d* = 0.67 and fewer structural attributions, *t*(264) = −2.99, *p* = .003, BCa 95% CI [−0.34, −0.06], *d = −*0.36. We also found support for Hypothesis 1b: Participants who perceived a person in persistent poverty considered them less deserving of receiving support than those who were assigned to the other condition, *t*(264) = 5.39, *p* < .001, BCa 95% CI [0.26, 0.53], *d* = 0.56. Our findings also confirmed Hypothesis 1c: Participants who perceived someone in persistent poverty showed a worse attitude towards social protection policies than participants who perceived someone in poverty due to economic crisis, *t*(264) = −5.23, *p* < .001, BCa 95% CI [−0.52, −0.24], *d = −*0.64. Table 4 presents the correlations between variables.

**Table 4:** Correlations Between Variables in Study 2

	1	2	3	4	5	6
1. Individualistic attributions	–					
2. Structural attributions	−.21**	–				
3. Deservingness of social protection	−.54**	.41**	–			
4. Social protection policies attitudes	−.55**	.27**	.60**	–		
5. Index identification	.09	−.01	.07	.16**	–	
6. Political ideology	.35**	−.18**	−.27**	−.45**	−.02	–

** *p* < 0.01 (2-tailed) * *p* < 0.05 level (2-tailed)

#### Group identification as moderator in the relationship among perceived group, deservingness of social protection, and attitudes towards social protection policies

**Fig. 1:**
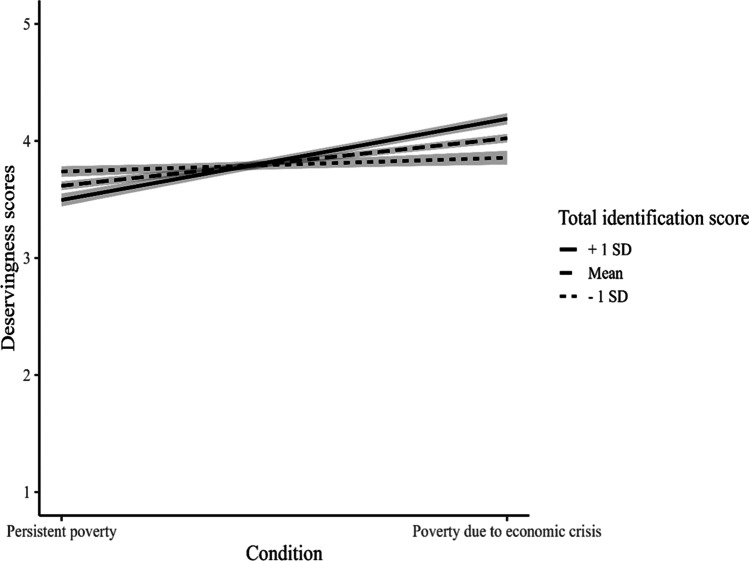
Identification with People in Poverty due to Economic Crisis as a Moderator Between Perceived Group and Deservingness Scores. Note: One standard deviation above the mean means more identification with those who are in poverty because economic crisis; one standard deviation below the mean means more identification with those in persistent poverty

We tested hypotheses H2 and H3 by conducting a linear regression analysis using the PROCESS macro for SPSS, testing moderation Model 1. Our results supported Hypothesis 3: Group identification moderated the relationship between perceived group and perceived deservingness of social protection, *b* = 0.26, *t*(262) = 3.71, *p* < .001, 95% CI = [0.12, 0.40]. Therefore, perceiving someone who is poor due to economic crisis (compared to someone in persistent poverty) increased deservingness perception among those who identified more with this group, *b* = 0.68, *SE* = 0.11, *p* < .001, 95% CI = [0.48, 0.89], but not among those who identified less with this group, *b* = 0.11, *SE* = 0.11, *p* = .28, 95% CI = [−0.51, 0.38] (see Fig. 1).

**Fig. 2:**
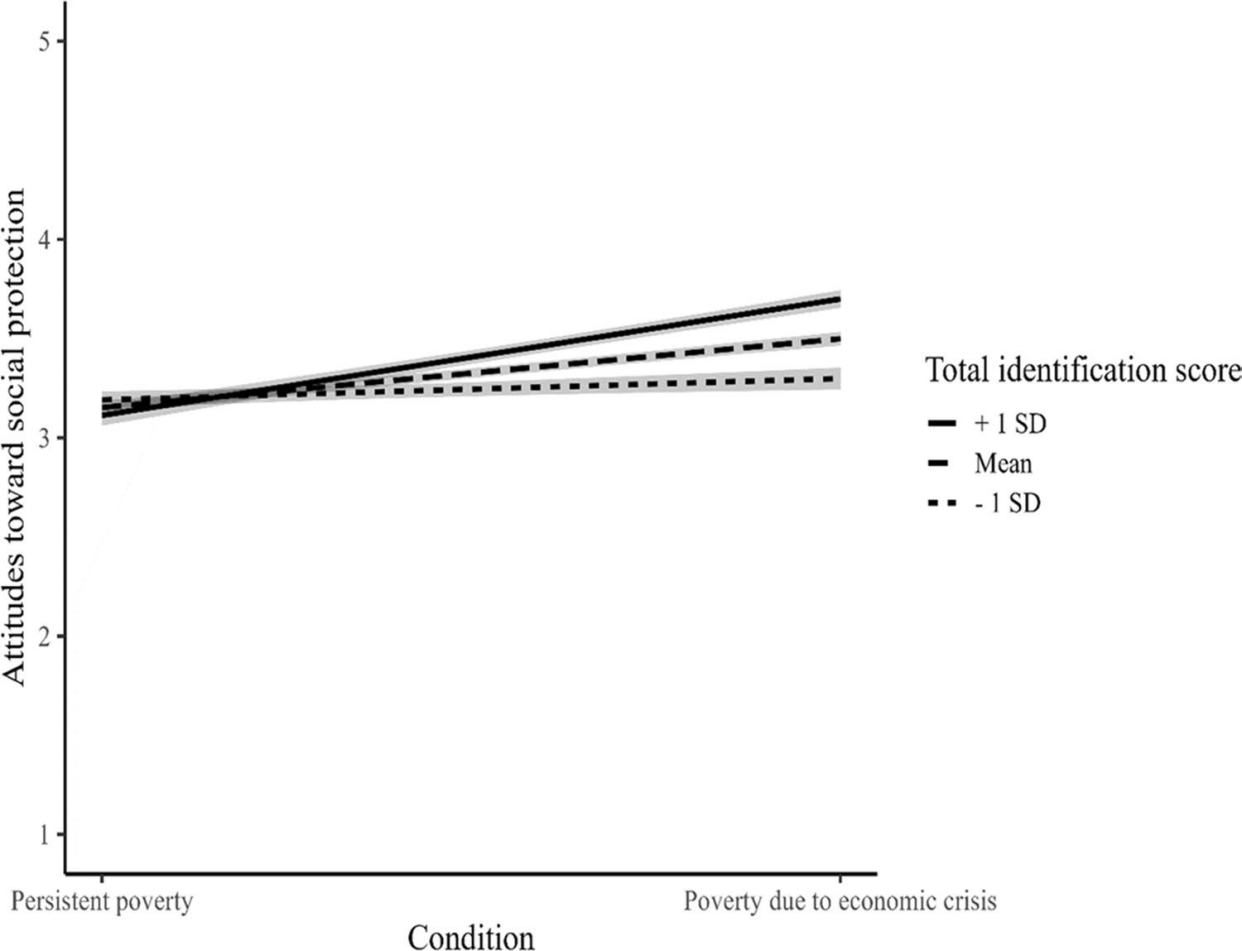
Identification With People in Poverty due to Economic Crisis as a Moderator Between Perceived Group and Attitudes Toward Social Protection Policies. Note: One standard deviation above the mean means more identification with those who are in poverty because economic crisis; one standard deviation below the mean means more identification with those in persistent poverty

As in Study 1, Hypothesis 3 was supported: Group identification moderated the relationship between perceived group and attitudes towards social protection policies, *b* = 0.22, *t*(262) = 3.29, *p* = .001, 95% CI = [0.09, 0.36]. As in Study 1, perceiving someone who is poor due to an economic crisis (compared to someone in persistent poverty) increased positive attitudes towards social protection policies among those who identified more with the first group, *b* = 0.58, *SE* = 0.10, *p* < .001, 95% CI = [0.35, 0.78], but not among those who identified less with this group, *b* = 0.11, *SE* = 0.10, *p* = .30, 95% CI = [−0.02, 0.35] (see Fig. 2).

#### The mediating role of attributions for poverty in the relationship among perceived group, deservingness of social protection, and attitudes towards social protection policies

**Fig. 3:**
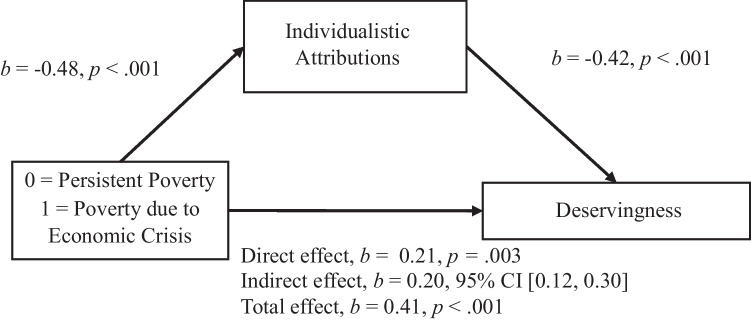
Individualistic Attributions as a Mediator Between Perceived Group and Deservingness Perception. Note: All confidence intervals for indirect effects are a BCa bootstrapped CI based in 1000 samples

**Fig. 4:**
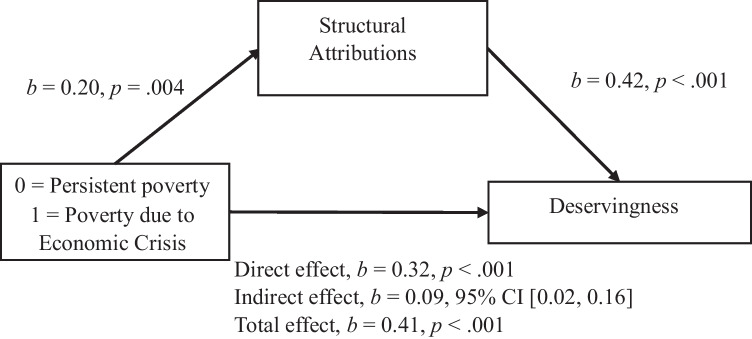
Structural Attributions as a Mediator Between Perceived Group and Deservingness Perception*.* Note: All confidence intervals for indirect effects are a BCa bootstrapped CI based in 1000 samples

We tested Hypotheses 4 and 5 by conducting a linear regression analysis using the PROCESS macro for SPSS, testing mediation Model 4. All confidence intervals for indirect effects were BCa CI based on 10,000 bootstrapped samples. The results supported Hypothesis 4: Structural and individualistic attributions mediate the relationship between perceived group and perceived deservingness of social protection (see Figs. 3 and 4). Regarding effect-size measures, *P*_*M*_ for structural attributions was 0.21, BCa 95% CI [0.06, 0.40], and *P*_*M*_ for individualistic attributions was 0.49, BCa 95% CI [0.31, 0.74].

The results fully support Hypothesis 5. We found a significant indirect effect of the perceived group on the attitudes towards social protection policiessocial-protection policies through individualistic attributions for poverty, *b* = 0.20, BCa 95% CI [0.12, 0.28]; *P*_*M*_ = 0.53, BCa 95% CI [0.33, 0.79]. In this model, the direct effect was *b* = 0.18, *p* = .006 and the total effect *b* = 0.38, p < .001; the perceived condition’s effect on individualistic attributions was *b* = −0.48, *p* < .001, and individualistic attributions’ effect on attitudes towards social protection policies was *b* = −0.41, *p* < .001. Structural attributions also played a statistically significant mediator role in this relationship, *b* = 0.05, BCa 95% CI [0.01, 0.10]; *P*_*M*_ = 0.13, BCa 95% CI [.04, .29]. In this model, the direct effect was *b* = 0.33, *p* < .001 and the total effect *b* = 0.38, *p* < .001; the perceived condition’s effect on structural attributions was *b* = 0.20, *p* = .004, and structural attributions’ effect on attitudes towards social protection policies was *b* = 0.24, *p* < .001. All the indirect effects described above remained significant when we controlled for political orientation.

**Fig. 5:**
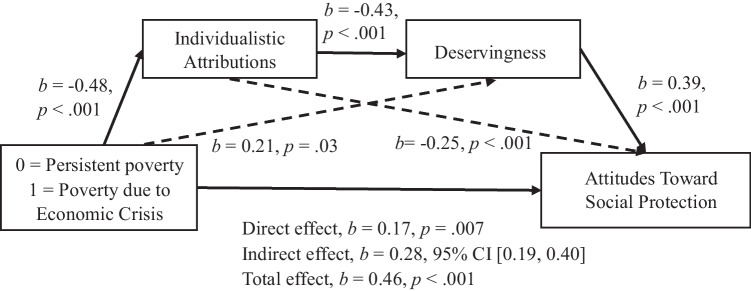
Model With Individualistic Attributions and Deservingness Perception as Mediators Between Perceived Group and Attitudes Towards Social Protection Policies. Note: All confidence intervals for indirect effects are a BCa bootstrapped CI based in 1000 samples. Condition 0 = persistent poverty; condition 1 = poverty due to economic crisis

In an exploratory regard, we tested a serial multiple-mediation model using the PROCESS macro for SPSS, Model 6, including attributions for poverty as the first mediating variable and deservingness of social protection as the second mediator. The independent variable was experimental condition, and the dependent variable was attitude towards social protection policies. This mediation model was also significant (see Fig. 5). We did not conduct the same analysis with structural attributions because considering the combined results of both studies, it seems that their role is not as relevant in this relationship. Finally, as in Study 1, using the lavaan package (Rosseel, 2012), we built a model including poverty attributions as parallel mediators, deservingness as a second serial mediator, condition as an independent variable, and attitudes towards social protection as a criterion in addition to group identification’s moderating effect (see Fig. S2 in Supplementary Materials). Individualistic and structural attributions remained significant mediators through social protection deservingness although, again, identification with the group ceased to play a significant moderating role.

### Discussion

Our findings from Study 1 were replicated in Study 2, and the hypotheses that were not confirmed in Study 1 are now confirmed. First, we found a different attitude towards social protection policies depending on the nature of the perceived target’s poverty. Again, the participants considered the person in persistent poverty more responsible for his situation and less deserving of help, and participants who perceived him showed a worse attitude towards social protection policies. Further, regarding people in poverty due to an economic crisis, this study supported our hypothesis (which was not supported in Study 1) that structural attributions for this poverty will lead to a better attitude towards social protection policies. The moderating effect of identification with people who are in poverty because of an economic crisis was replicated, but, again, it ceased to play a significant role as a moderator when we included all mediating variables in the same model. Interestingly, we found support for our double-mediation model: The type of person in poverty influences the attributions for his poverty, which influence the target’s deservingness of social protection, which influences the attitudes towards social protection.

## Meta-analysis of studies 1 and 2

Following the recommendations Goh et al. (2016) made regarding meta-analysis, we conducted a meta-analysis with fixed effects, including measures present in both studies, analysing perceived condition’s overall effect on individualistic attributions, structural attributions, and attitudes towards social-protection policies. We did not preregister this analysis; therefore, it should be understood as exploratory. First, we transformed Cohen’s *d* into Pearson’s correlation coefficients and calculated the weighted means of correlations, previously transforming correlations coefficients into Fisher’s *z* for normalization. Then, we combined *r*_z_ values meta-analytically using the formula Goh et al. (2016) provided. We transformed Fisher’s *z* values into Pearson’s correlation coefficients and the latter into Cohen’s *d* values to facilitate interpretation of the results. Moreover, we aggregated *p* values using Fisher’s method using the “aggregation” (Yi & Pachter, 2018) R Package (R Core Team, 2017). Considering both studies described in this paper, people in persistent poverty, compared to those in poverty due to an economic crisis, received more individualistic attributions, *d* = 0.48, *p* < .001, and less structural attributions, *d* = 0.24, *p* = .01. Participants also showed better attitudes towards social protection policies when they perceived the latter, *d* = 0.54, *p* < .001.

To test mediation effects across both studies, we performed an integrative data analysis (Curran & Hussong, 2009). Mediation analysis revealed a significant indirect effect of the group perceived on the attitudes towards social protection policies through individualistic attributions for poverty, *b* = 0.14, BCa 95% CI [0.09, 0.20]; *P*_*M*_ = 0.47, BCa 95% CI [0.31, 0.66]. In this model, the direct effect was *b* = 0.16, *p* < .001 and the total effect *b* = 0.31, *p* < .001; perceived condition’s effect on individualistic attributions was *b* = −0.34, *p* < .001, and individualistic attributions’ effect on attitudes towards social protection policies was *b* = −0.42, *p* < .001. Considering both studies, structural attributions also significantly mediated the relationship between perceived group and attitudes towards social protection policies, *b* = 0.02, BCa 95% CI [0.00, 0.04]; *P*_*M*_ = 0.07, BCa 95% CI [0.01, 0.16]. In this model, the direct effect was *b* = 0.29, *p* < .001 and the total effect *b* = 0.31, *p* < .001; the perceived condition’s effect on structural attributions was *b* = 0.13, *p* = .007, and structural attributions’ effect on attitudes towards social protection policies was *b* = 0.16, *p* = .001. These indirect effects remained significant even after we controlled for political orientation.

These analyses provide evidence of the robustness of the effect of individualistic poverty attributions on the relationship between perceived group of people in poverty and attitudes towards social protection policies. However, the case is more complex regarding structural attributions, as we found significant effects. Indirect effect and effect size showed small values, raising concerns about their importance in the relationship mentioned above.

## General discussion

These two studies show that when someone who has always been in poverty is perceived, people’s general attitudes towards social protection policies are worse than when they perceive someone who is in more circumstantial poverty, such as that caused by an economic crisis. Our study gives us some clues about how this could happen. People perceive those in persistent poverty as more responsible for their situation and less deserving of help, which leads to a worse attitude towards social protection policies. People in persistent poverty are also perceived as less competent than those who are poor due to an economic crisis. These results are stronger in the second study, which we conducted with a sample drawn from the general population. Although in our other study we had already studied the differences in perception between different subgroups of people with few resources (e.g., Fiske et al., 2007) and how different poverty types affect attitudes towards public policies (Henry et al., 2004), as far as we know, this is the first study comparing these two specific subgroups.

Regarding attributions, our results show that individualistic attributions for poverty are important in the perception of both types of people in poverty and in the attitudes towards social protection policies for these groups, considering that people in persistent poverty are considered more responsible for their situation, which leads to a worse attitude towards social protection policies. This result makes sense given previous research on how different groups of people with few resources evoke different levels of support for social protection policies (Alston & Dean, 1972; Bullock et al., 2003). Regarding structural attributions, we found a significant effect of these attributions on attitudes towards social protection policies in Study 2 but not in Study 1. The analysis of the results of both studies combined suggests that the effect is significant but small and not of great relevance in explaining how poverty type influences attitudes towards social protection policies.

Determining attributions’ influence on social policies, however, can be a somewhat complex task. Although we distinguished individualistic from structural attributions, this division may pose some problems. For example, in our research, we considered attributions to ability an individualistic cause, but we do not know if people perceive this cause as something totally dependent on the person or also as a consequence of society (e.g., they may not have capacity because they have not received a “good” education, or they may not want to move to other places to work because they lack the resources to do so; for a critique, see Lepianka et al., 2009). We can say the same about the lack of savings. It is basically an individualistic cause but one that can also be perceived as structural (a consequence of the education received; for a critique, see Lepianka et al., 2009). Therefore, in the second study, we introduced a complementary measure of deservingness perceptions of social protection to determine whether those who participated in our study believe that the types of people in poverty considered deserve to receive support from institutions. This also allowed us to verify the direct relationship between the attributions, this deservingness and the general support for social protection policies. The results show that people who have always been poor are perceived as less deserving of help than those who are poor due to an economic crisis. Specifically, the participants considered those in the first group less needy and had a worse attitude towards those receiving help and less able to offer reciprocity. In addition, our results show that individualistic attributions are closely related to the deservingness of help that the person belonging to each group of people in poverty is believed to have. Specifically, the mediation carried out in Study 2 shows that perceiving people in persistent poverty leads to more individualistic attributions, and this perception leads to less deservingness of social protection and the latter to have worse attitudes towards social protection policies. Therefore, our measures of attribution could be questioned. However, the participants clearly believed those causes considered individualistic in our research imply lower deservingness of receiving help and attributed these causes more frequently to the persistently poor. This finding also fits with the previous literature on how deservingness judgements affect attitudes towards social protection policies (van Oorschot, 2000, 2006; Will, 1993).

We must also bear in mind that our results show that the participants’ identification with each of the groups of poor can influence the results. Perceiving someone who is poor due to an economic crisis (in comparison to someone in persistent poverty) increased deservingness perception of social protection among those who identified more with this group but not among those who identified less with this group.

Somewhat surprisingly, stereotypes about people in poverty, at least measured according to the stereotype-content model (Glick & Fiske, 1999), did not have a significant effect on the relationship between perceived group and attitudes towards social protection policies. Although we found that people in persistent poverty are perceived as less competent, this perception does not seem to explain the worse attitude towards social protection policies. This may be because we are nonetheless comparing two groups of people in poverty and the differences in these stereotypes between both groups are not so large.

The effects of the 2008 economic crisis have been terrible for much of the world’s population, and now these effects can join those caused by the COVID-19 crisis. The massive loss of employment in the wake of the economic crisis led many people to become declassed, facing difficulties they had not previously known. Although this striking new reality—and its psychosocial effects on people—justifies the topic’s relevance in empirical research, we run the risk of neglecting another disadvantaged group that regrettably has always existed in most societies: people in persistent poverty.

Recent research has highlighted the importance of dehumanization processes towards people living in poverty in attitudes towards poverty alleviation policies (Sainz et al., 2019, 2020a) and the mediation role of attributions for poverty in this relationship (Sainz et al., 2020b). Our study offers an opportunity to determine whether the processes of dehumanization of people in poverty are differentiated based on the group in question as well as how this differentiation may influence attitudes towards social protection. Other studies have shown that enhancing structural attributions for poverty can lead to more egalitarian attitudes and less support for inequality (Piff et al., 2020). Our results suggest that another related factor to take into account when planning actions to improve these attitudes may be the type of poverty we are talking about. Another important line of future research could be to examine how contact with each of the two groups influences attitudes towards social protection and the way in which attributions for poverty vary. Contact with inequality in everyday life has been shown to influence attitudes towards redistribution (García-Castro et al., 2020), and this inequality is perceived through indicators such as consumption and opportunities (García-Castro et al., 2022). Given that different groups in poverty may have different consumption patterns, leisure habits, opportunities, and contact with people from other economic groups, this perception of inequality in everyday life may be an important factor in the relationship we examined.

Our studies present some limitations. First, the samples in both studies have some potential weaknesses. The sample in Study 1 comprised undergraduate students, which may affect the results’ generalizability, especially for a topic such as the one we addressed (e.g., Wintre et al., 2001). The sample in Study 2 comprised participants from the general population. This sample added variability in terms of social background, but we recruited them in a public-transport station, so they might not represent the entire population. Nevertheless, the results of the mini meta-analysis showed consistency in the patterns we found. Second, the protagonist of our manipulation was a man, and we know that men and women in poverty can be perceived differently (Cozzarelli et al., 2002). Therefore, it would be interesting to test in future research whether our findings are replicated when the perceived target is a woman. Third, although the mediation process we propose is theoretically grounded, other relevant variables may influence this relationship between type of poverty and attitudes towards social-protection policies. Fourth, although our measures have shown acceptable internal consistency in both studies and have correlated with other variables as expected according to previous literature, they have not followed a process of adaptation and validation in Spanish as such. Future research on the adaptation of these measures could strengthen the validity of the results obtained. Finally, another limitation arises from the fact that we conducted these studies in 2019, before the emergence of the global threat of COVID-19. This fact does not diminish our findings’ significance, but it raises new questions about the object of study, as we know that recognizing COVID-19’s impact shifts attributions for poverty (Wiwad et al., 2020).

The greater responsibility attributed to the persistently poor and its effects on attitudes towards social protection policies could also mean that their needs do not become part of the priorities of the political sphere that makes decisions about resource distribution. This is a presupposition that should be tested in future studies, but the previous literature on how public perception influences political decisions suggests that it may be something to consider (Brooks & Manza, 2006). The process described above may be an important mechanism in the perpetuation of poverty.

## Supplementary Information

ESM 1 (DOCX 96 kb)

## Data Availability

All measures, datasets, and preregistration forms for our studies can be found at Open Science Framework repository: https://osf.io/ne6ug/?view_only=e0a2dd15b1c4495b95c527df89569038.

## Declarations

This research has been conducted conform to the American Psychological Association’s Ethical Principles in the Conduct of Research with Human Participants. Participants were asked for their informed consent before starting the surveys.

### Open practices

Data, scripts for the main analyses, measures, and pre-registration forms of both studies are publicly available at: https://osf.io/ne6ug/?view_only=af98fb36c3ef4eb98fa19542cde3467b
