# Supplementary material for: Not all poor are equal: the perpetuation of poverty through blaming those who have been poor all their lives
Source: Curr Psychol. 2022 Oct 5:1–17. Online ahead of print. doi: 10.1007/s12144-022-03804-6 (PMC9533286; doi:10.1007/s12144-022-03804-6)
Supplement: Supplementary file 1 — (DOCX 96 kb) [file 12144_2022_3804_MOESM1_ESM.docx]

**Study 1**

**All measures used**

**Social Dominance Orientation** (α = .87). This variable was measured using the Social Dominance Orientation Scale (Pratto et al., 1994), validated and adapted to Spanish by Silván-Ferrero and Bustillos (2007). This scale has 16 items (e.g., “Some groups of people are just more worthy than others,” “No one group should dominate in society”) with a 1 (Totally disagree) to 7 (Totally agree) Likert response.

**System Justification Scale** (α = .82)*.* Measured through a translation and adaptation of the System Justification Scale (Kay & Jost, 2003). The adaptation consisted of adapting the wording of the items to the Spanish context. For example, where the original scale says "United States is the best country to live in" we changed the item to: "Spain is the best country to live in ". It was composed of eight items (e.g., “In general, I find society to be fair”) with a Likert response format of 1 (Totally disagree) to 8 (Totally agree).

**Economic Threat** (α = .89)*.* We used the Financial Threat Scale (Marjanovic et al., 2013), translated to Spanish. This scale consisted of five items (e.g., “How economic/financial uncertainty do you feel?”) with a 1 (Totally disagree) to 5 (Totally agree) Likert response.

In addition, we measured social class in the following way:

1. Subjective social class: MacArthur Scale of Subjective SES (e.g., Kraus et al., 2009). It consisted of a ladder with 10 rungs representing people with different levels of education, income, and occupational status. Participants placed a mark on the rung where they feel they stand relative to the society.

2. Objective social class: Objective social class was measured based on scores on income and participants’ educational level. These scores were standardized and summed to obtain a general standardized measure of objective SES (e.g., Piff et al, 2010; Navarro-Carrillo et al., 2018).

**All the results of the hypotheses in the pre-registration**

1. The scores in competence and warmth attributed to the target will show a negative relationship with scores in the system justification scale and scores in social dominance scale.

There is not a statistical significant relation. Warmth scores did not correlate with social dominance orientation (*r* = -.07, *p* = .29) and system justification (*r* = -.01, *p* = .93). Competence scores did not correlate with social dominance orientation (*r* = -.06, *p* = .38) and system justification (*r* = -.02, *p* = .79).

2. Favourable attitudes towards social protection policies will correlate negatively with scores in the system justification scale and scores in social dominance scale.

We found a significant relation between attitudes towards social protection policies and social dominance orientation (*r* = .40, *p* < .001) and system justification (*r* = .41, *p* < .001).

3. Social class will moderate the relation between the perceived group –person in chronic poverty (coded 0) vs poor person because of economic crisis (coded 1)- and the assignment of competence and warmth.

We did not found a significant moderator effect of subjective or objective social class.

4. Social class will moderate the relation between the perceived group –person in chronic poverty (coded 0) vs poor person because of economic crisis (coded 1)- and the identification with the group.

An effect of perceived group x Subjective SES on identification with the group was found, *b* = -0.20, *t*(248) = -2.57, *p* = .01. 95%CI = [-0.36, -0.05]. When perceiving a person in poverty due to economic crisis (in comparison with someone in persistent poverty), identification with them increased—but only among low subjective SES participants, *b* = 0.25, *SE* = 0.12, *p* = .037, 95%CI = [0.01, 0.50]; however, among those with higher subjective SES the effect was non-significant, *b* = -0.14, *SE* = 0.14, *p* = .3, 95%CI = [-0.42, 0.13]. Objective SES did not have a statistically significant effect.

5. The relation between the perceived group (people who is poor because of economic crisis vs. people in chronic poverty) and the assignment of scores in competence and warmth will be mediated by causal attributions on poverty.

We did not found support for this hypothesis.

6. The relationship between the perceived group (people who is poor because of economic crisis vs. people in chronic poverty) and the assignment of scores in competence and warmth will be mediated by the financial threat perception.

We did not found support for this hypothesis.

**Table S1.**

*Correlations Between all Variables in Study 1*

|  |  | 1 | 2 | 3 | 4 | 5 | 6 | 7 | 8 | 9 | 10 | 11 | 12 |
| --- | --- | --- | --- | --- | --- | --- | --- | --- | --- | --- | --- | --- | --- |
| 1.Social Dominance Orientation |  | 1 |  |  |  |  |  |  |  |  |  |  |  |
| 2.System Justification |  | .51^**^ | 1 |  |  |  |  |  |  |  |  |  |  |
| 3. Competence |  | -.06 | -.02 | 1 |  |  |  |  |  |  |  |  |  |
| 4. Warmth |  | -.07 | -.01 | .65^**^ | 1 |  |  |  |  |  |  |  |  |
| 5. Individualistic attributions |  | .38^**^ | .44^**^ | -.03 | -.03 | 1 |  |  |  |  |  |  |  |
| 6. Structural attributions |  | -.15^*^ | -.19^**^ | .11 | .03 | -.07 | 1 |  |  |  |  |  |  |
| 7. Attitudes toward social protection |  | -.40^**^ | -.41^**^ | .00 | .08 | -.57^**^ | .05 | 1 |  |  |  |  |  |
| 8. Political ideology |  | .37^**^ | .47^**^ | .01 | -.01 | .41^**^ | -.04 | -.59^**^ | 1 |  |  |  |  |
| 9. Financial threat |  | -.13^*^ | -.26^**^ | .03 | .02 | -.14^*^ | -.01 | .24^**^ | -.22^**^ | 1 |  |  |  |
| 10. Total identification index |  | .11 | .15^*^ | -.08 | .01 | .07 | -.16^*^ | .12 | .03 | .18^**^ | 1 |  |  |
| 11. Objective social class |  | .01 | .11 | -.13^*^ | -.13^*^ | -.05 | -.01 | .02 | .03 | -.31^**^ | .01 | 1 |  |
| 12. Subjective social class |  | .13^*^ | .20^**^ | .00 | -.01 | .01 | -.05 | -.09 | .27^**^ | -.43^**^ | .07 | .39^**^ | 1 |

** *p* < 0.01 (2-tailed). * *p* < 0.05 (2-tailed).

**Figure S1.**


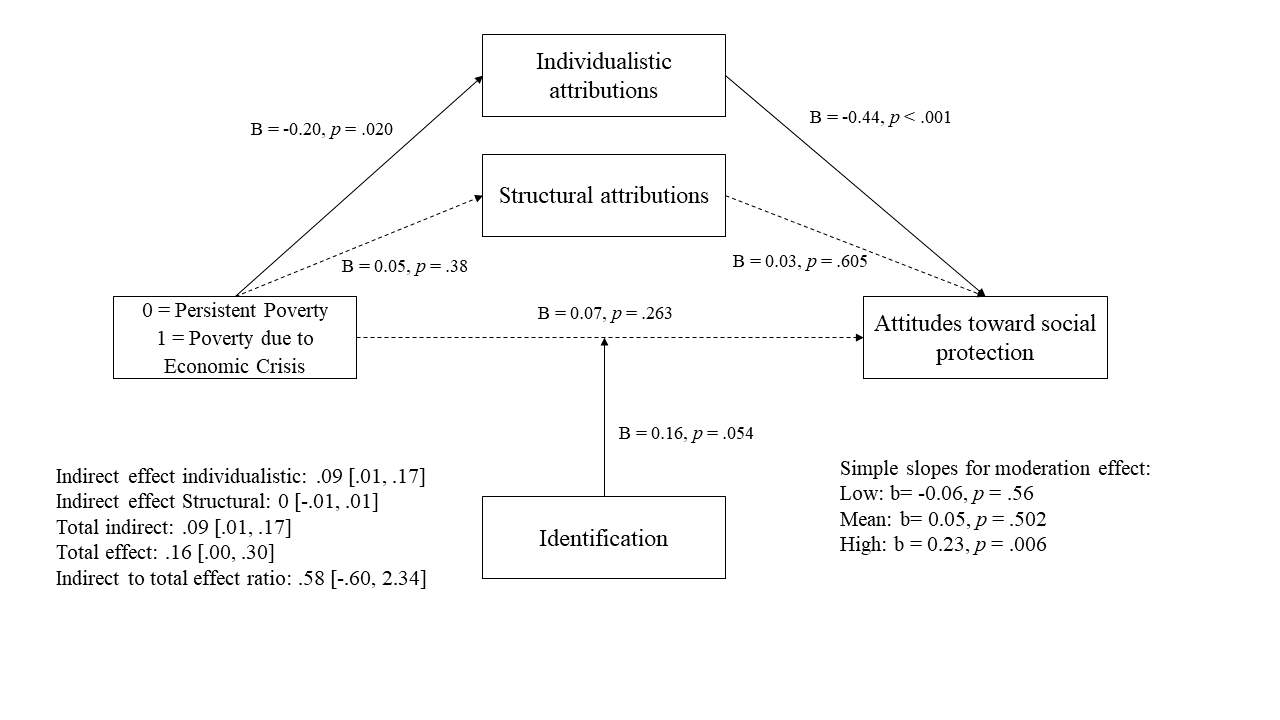
*Model Including the Mediation Effects of Attributions and the Moderation Effect of Identification with the Group.*

**Study 2**

**All measures used**

**Social Dominance Orientation** (α = .88). This variable was measured in the same way as in Study 1.

Sociodemographic and social class information was collected as in Study 1.

**All the results of the hypotheses in the pre-registration**

1. The scores in deservingness attributed to the target will show a negative relationship with scores in the social dominance orientation scale.

This hypothesis was confirmed (*r* = -.47, *p* < .001).

2. Favourable attitudes towards social protection policies will correlate negatively with scores in social dominance orientation scale.

This hypothesis was confirmed (*r* = -.55, *p* < .001).

3. Social class will moderate the relation between the perceived group –person in chronic poverty (coded 0) vs poor person because of economic crisis (coded 1)- and the identification with the group.

Neither objective nor subjective socioeconomic status moderated the relation between perceived group and identification with the group.

Table S2. Correlations Between all Variables in Study 2

|  | 1 | 2 | 3 | 4 | 5 | 6 | 7 | 8 | 9 |
| --- | --- | --- | --- | --- | --- | --- | --- | --- | --- |
| 1. Social Dominance Orientation | 1 |  |  |  |  |  |  |  |  |
| 2. Individualistic attributions | .43^**^ | 1 |  |  |  |  |  |  |  |
| 3. Structural attributions | -.23^**^ | -.21^**^ | 1 |  |  |  |  |  |  |
| 4. Deservingness | -.047^**^ | -.54^**^ | .41^**^ | 1 |  |  |  |  |  |
| 5. Attitudes toward social protection | -.54^**^ | -.55^**^ | .27^**^ | .60^**^ | 1 |  |  |  |  |
| 6. Total identification index | -.09 | .09 | -.01 | .07 | .16^**^ | 1 |  |  |  |
| 7. Political ideology | .44^**^ | .35^**^ | -.18^**^ | -.27^**^ | -.45^**^ | .02 | 1 |  |  |
| 8. Objective social class | .01 | .02 | -.09 | -.02 | .14^*^ | .09 | -.04 | 1 |  |
| 9. Subjective social class | .10 | .05 | -.01 | -.01 | -.08 | .03 | .25^**^ | .09 | 1 |

** *p* < .01 (2-tailed). * *p* < .05 (2-tailed).

**Figure S2.**


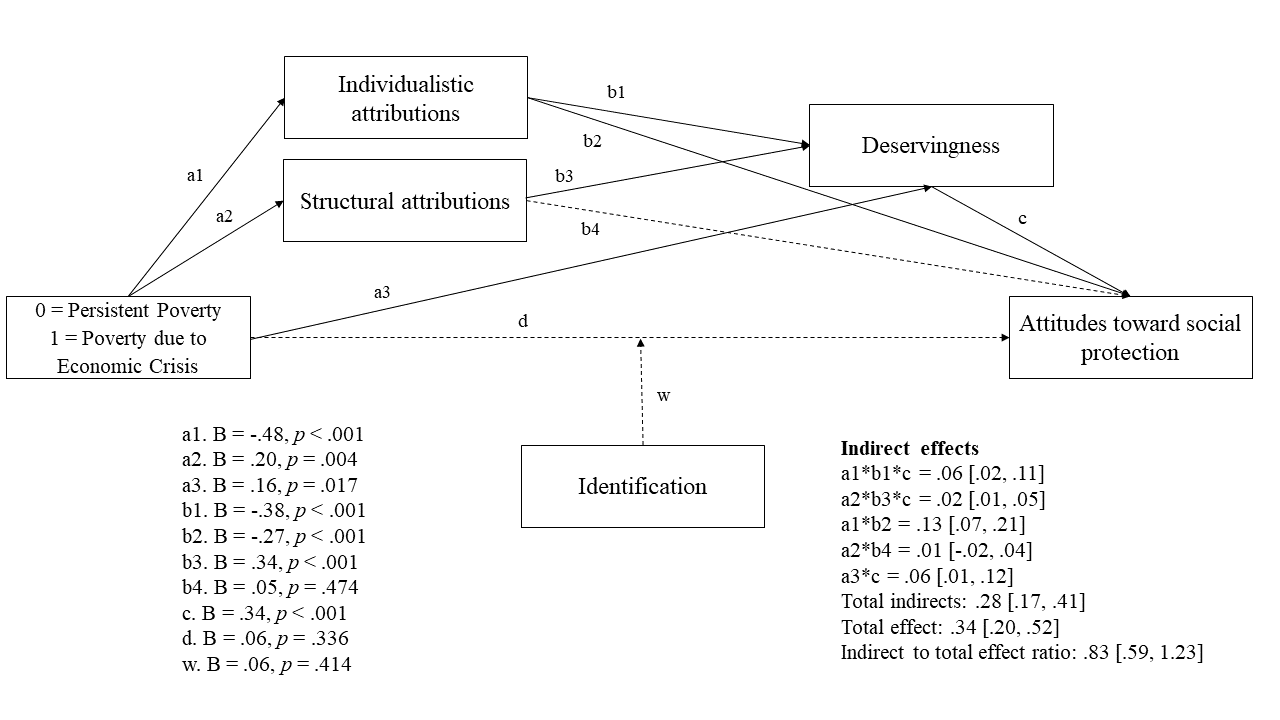
*Model Including the Mediation Effects of Attributions and Deservingness of Social Protection and the Moderation Effect of Identification with the Group.*

**References**

Kay, A. C., & Jost, J. T. (2003). Complementary justice: effects of “poor but happy" and "poor but honest" stereotype exemplars on system justification and implicit activation of the justice motive. *Journal of Personality and Social Psychology*, *85*(5), 823-837. [https://doi.org/1.1037/0022-3514.85.5.823](https://doi.org/10.1037/0022-3514.85.5.823)

Kraus, M. W., Piff, P. K., & Keltner, D. (2009). Social class, sense of control, and social explanation. *Journal of Personality and Social Psychology*, *97*(6), 992-1004. [https://doi.org/1.1037/a0016357](https://doi.org/10.1037/a0016357)

Marjanovic, Z., Greenglass, E. R., Fiksenbaum, L., & Bell, C. M. (2013). Psychometric evaluation of the Financial Threat Scale (FTS) in the context of the great recession. *Journal of Economic Psychology*, *36*, 1-1. [https://doi.org/1.1016/j.joep.2013.02.005](https://doi.org/10.1016/j.joep.2013.02.005)

Navarro-Carrillo, G., Valor-Segura, I., & Moya, M. (2018). Do you trust strangers, close acquaintances, and members of your ingroup? Differences in trust based on social class in Spain. *Social Indicators Research*, *135*(2), 585-597. [https://doi.org/1.1007/s11205-016-1527-7](https://doi.org/10.1007/s11205-016-1527-7)

Piff, P. K., Kraus, M. W., Côté, S., Cheng, B. H., & Keltner, D. (2010). Having less, giving more: the influence of social class on prosocial behavior. *Journal of Personality and Social Psychology*, *99*(5), 771-784. [https://doi.org/1.1037/a0020092](https://doi.org/10.1037/a0020092)

Pratto, F., Sidanius, J., Stallworth, L. M., & Malle, B. F. (1994). Social dominance orientation: A personality variable predicting social and political attitudes. *Journal of Personality and Social Psychology*, *67*(4), 741-763. [https://doi.org/1.1037/0022-3514.67.4.741](https://doi.apa.org/doi/10.1037/0022-3514.67.4.741)

Silván-Ferrero, M. D. P., & Bustillos, A. (2007). Adaptación de la Escala de Orientación a la Dominancia Social al castellano: validación de la dominancia grupal y la oposición a la igualdad como factores subyacentes. *Revista de Psicología Social*, *22*(1), 3-15. [https://doi.org/1.1174/021347407779697485](https://doi.org/10.1174/021347407779697485)
